# Early intervention and follow-up programs among children with cerebral palsy in Moldova: potential impact on impairments?

**DOI:** 10.1186/s12887-020-1931-7

**Published:** 2020-01-22

**Authors:** Ecaterina Gincota Bufteac, Guro L. Andersen, Larisa Spinei, Reidun Birgitta Jahnsen

**Affiliations:** 1 CEI Voinicel - Center of Early Intervention Voinicel, Chisinau, Moldova; 2 Oslo Metropolitan University, Oslo, Norway; 3 Cerebral Palsy Registry of Norway, Vestfold Hospital Trust, Tonsberg, Norway; 4 Norwegian University of Science and Technology, Trondheim, Norway; 5 State University of Medicine and Pharmacy Nicolae Testimitanu, Chisinau, Moldova; 6 Oslo University Hospital and University of Oslo, Institute of Health and Society, CHARM, Oslo, Norway

**Keywords:** Cerebral palsy, Moldova, Subtype, Severity, Associated impairments, Contractures, Early intervention services, Follow-up program

## Abstract

**Aim:**

To study whether early intervention services (EI) and a follow-up program (FU) influence outcomes of children with cerebral palsy (CP) in Moldova.

**Methods:**

Records from 351 children with CP in Moldova born during 2009 and 2010 were retrieved from hospital and orphanage archives between 1 July 2016 and 30 September 2017. We investigated the proportion enrolled in EI and FU at the Early Intervention Centre Voinicel and at the Institute of Mother and Child in 2009–2012. Logistic regression analyses were applied to calculate crude and adjusted odds ratios (OR) with 95% confidence intervals (CI) for outcomes in children enrolled and not enrolled.

**Results:**

Among all children with CP, 166 (47%) were enrolled in EI and FU. Of the 51 children born extremely preterm (gestational age ≤ 31 weeks), 46 (90%) were enrolled, compared to 97 (39%) of the 250 children born at term. Among 110 non-walking children with CP, 82 (74%) were enrolled into EI and FU, compared to 84 (35%) of 241 able to walk.

There was no difference in outcomes of cognition, communication, vision and hearing impairments between those enrolled or not enrolled in EI and FU. However, the subgroup analyses showed that the risk of contractures was 11 times higher among non-walking children who were not enrolled in EI and FU programs (OR = 10.931, 95% CI 2.328–51.328, *p* = 0.002).

**Conclusion:**

In Moldova, EI and FU seem to be offered mostly to extremely preterm and non-walking children with CP. The results indicate a decreased risk for contractures in these children.

## Background

Cerebral palsy (CP) is an umbrella term covering disorders of movement and posture, leading to activity limitations, caused by non-progressive lesions or malformations in the immature brain [1]. Associated disturbances of sensation, perception, cognition, communication and behaviour are frequent, as are secondary musculoskeletal problems and epilepsy [1]. The brain lesion is non-progressive, but its consequences do change over time, and can be barriers to activity and participation in different life arenas [2].

In all children the most rapid maturation and development of both the musculoskeletal and central nervous systems occurs during preschool age, and this development forms the basis for future function [3]. Among clinicians in paediatric rehabilitation, a general consensus is that children with lesions of the central nervous system should receive rehabilitation interventions as soon as possible, due to the rapid brain development during early life [4].

Clinicians and researchers therefore share a common goal to enhance development of function and prevent known secondary complications in as many developmental areas as possible in children at risk of CP, or already diagnosed with CP. These services are called early intervention (EI). EI is differently defined across countries, and there is no consensus regarding age. The Early Intervention Handbook [5] defines it as “multidisciplinary services provided to children from birth to five years of age to promote child health and well-being, enhance emerging competencies, minimise developmental delays, remediate existing or emerging disabilities, prevent functional deterioration and promote adaptive parenting and overall family function”.

EI includes several rehabilitation services targeting five developmental areas (cognitive, physical, communication, adaptive and social–emotional). Early life is the period of the highest developmental potential due to the high plasticity of the immature brain [6]. A review by De Graaf-Peters and Hadders-Algra [6] suggested that intervention prior to 40–44 weeks post menstrual age (PMA) should be restricted to interventions aiming to mimic the intrauterine environment, such as the Newborn Individualized Developmental Care and Assessment Program (NIDCAP). However, after 40–44 weeks, the intervention should include active stimulation of the child’s development. Recent research has documented that the period of dendritic outgrowth and active synapse formation is the best period for the repair of brain damage. This indicates that the period between 28 weeks PMA and 15 months postnatally would be best for EI [6].

However, a recent systematic review by Morgan et al. concluded that the evidence for early motor intervention is limited by the lack of high-quality trials [7]. The most promising intervention included child-initiated movement, task specificity and environmental modification [7]. This systematic review showed the importance of family-centred activities as well as specific environmental changes.

Guralnic [8] also described the positive effect on mother-child interaction of an EI program for both preterm and term-born children. Another study showed a positive effect of a Mother-Infant Transaction Program (MITP) on important qualities of social interaction between mothers of moderately and late preterm infants at 12 months. Being a first-time mother seems to be a mediator that enhances the effects of the intervention [9].

A recent Cochrane review by Spittle et al. [10] concluded that EI in infants born preterm (< 37 gestational weeks) is associated with improved cognitive development during infancy and preschool age, and a minor positive effect on infant motor development.

Interestingly, the general positive effects of EI occur in the presence of a large variety of theoretical concepts and actual program content. Parent–infant relationships have a greater impact on cognitive outcomes at infancy and preschool age than intervention programs that focus on either infant development or parent support [10].

Fewer studies examine the effect of EI on children born at term with CP. The available results among children with CP born at term showed a large variation in intervention approaches, similar to EI in infants born preterm [10]. According to Novak, the preferable interventions are child-active approaches that induce maximal neuroplasticity [4]. Moreover, specific motor training programs, such as training locomotor movements on a treadmill and general developmental programs, targeting the child’s exploration of active motor behaviour, showed a positive effect on motor development [11].

Early detection of children with CP or at high risk of developing CP is crucial to including them as soon as possible in EI programs. Predictive tools for CP are now available, such as the Hammersmith Infant Neurological Evaluation (HINE), the General Movement Assessment (PRECHTL GMA) tool, and cerebral MRI classifications [4].

High-quality evidence to support EI in improving neurodevelopmental outcomes is sparse. The existing evidence for infants with CP or at high risk of CP recommends interventions based on motor learning principles, active involvement of the parents and enrichment of the environment [4, 7, 10].

### Setting – the situation in Moldova

In 2003, the first Centre of Early Intervention (CEI) Services Voinicel was founded in Chisinau, Moldova, with the help of the Norwegian non-governmental organisation Ahead-Moldova. The need for family-based intervention was identified in order to address the high rate of abandoned children with disabilities in four orphanages, resulting from a lack of services for families with children at risk of developmental disorders, as well as those with identified disabilities.

The National Agency of Public Health has reported that neurological disability is the second most frequent paediatric disability in Moldova, and that 60% of those with a neurological disability are children with CP [12]. Therefore, some early-stage interventions had to be implemented as soon as possible.

In 2008, a study of the multidisciplinary model of Early Intervention (EI) services for children with disabilities in Moldova was performed, resulting in a permanent EI program [13].

Around the same time that CEI Voinicel began providing EI services, neonatal mortality and morbidity prevention strategies in Moldova were established in three national programs [14].

The Concept of Neonatal Diagnosis and Surveillance Service [14] was elaborated and implemented during 2008 for the care of very low birthweight children (< 1500 g), based on the same decision as the Ministry of Health no. 118 of 20 February 2009 [14], which lead to implementation of follow-up program (FU) providing screening, diagnostics and rehabilitation services for children at risk (0–3 years old) at the Institute of Mother and Child in Chisinau.

The aim of this study was to explore whether EI and FU programs potentially could influence impairment outcomes in children with CP, by comparing impairment outcomes in those enrolled and those not enrolled in EI and FU programs.

## Methods

### Design and study population

The study is a retrospective cohort study comparing impairment outcomes in children with CP who had been enrolled into an early intervention and/or follow-up program and children with CP not receiving early intervention or follow-up.

**Fig. 1:**
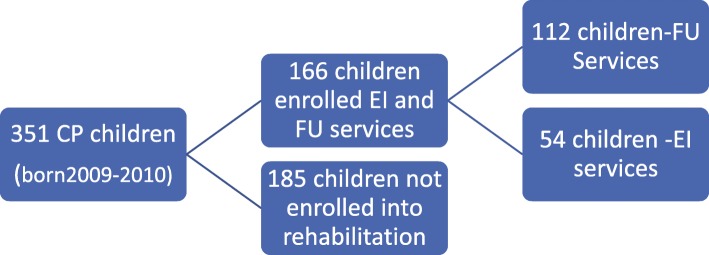
Flow-chart of the included study sample

Eligible for inclusion in this comparative cross-sectional study were children with CP enrolled in FU from 2009 to 2012 at the National Hospital Institute of Mother and Child, children enrolled in EI at CEI Voinicel, and children with CP not enrolled in rehabilitation programs, born between 1 January 2009 and 31 December 2010 (Fig. 1). These two clinics were the only ones providing early intervention services at this time. The National Hospital Institute of Mother and Child is the reference hospital for children with disabilities living outside the capital area, covering approximately 2.7 million (75%) of Moldova’s 3.5 million inhabitants, all children with severe neurological diagnosis are referred to this hospital, while CEI Voinicel covers the population of 670,000 within the Chisinau municipality.

Children diagnosed with CP were identified by the first author via medical records. Detailed information was identified by scrutinizing the medical records of each child from several departments of the Institute of Mother and Child and from the CEI Voinicel database. Antenatal and perinatal information was collected from medical records at the maternity ward, the Departments of Neonatal Care and Premature Birth, and the Neonatal Intensive Care Unit (NICU), which were the existing centres at the time of the study.

Having the initial complete information and the data extracted from the two above mentioned clinics, we had the opportunity to make comparison of impairment outcomes of the children enrolled in early intervention and/or follow-up program and those who were not.

### Clinical characteristics – outcome measures

CP was diagnosed and classified according to the Surveillance of Cerebral Palsy in Europe (SCPE) protocol [2], into spastic, dyskinetic, ataxic and not classified subtypes. The spastic subtype was sub-classified into uni- and bilateral CP, spastic unilateral CP into right and left hemiplegia, and spastic bilateral CP into quadriplegia and diplegia, in accordance with the International Classification of Diseases 10th revision (ICD 10) [15].

Gross motor function was classified according to the Gross Motor Function Classification System (GMFCS) [16]. As the data were collected from descriptions in medical records of variable quality, the children’s gross motor function was dichotomised into walking (GMFCS levels I, II and III), and non-walking (GMFCS levels IV and V) children, as proposed by Andersen et al. [17] and used in a previous study by Gincota et al. [18].

Fine motor function was classified according to the Bimanual Fine Motor Function (BFMF) scale [19] and was described by speech therapists as free text in medical records, as the development of communication at an early stage is closely related to use of hands, and dichotomised into BFMF level I–III, and BFMF levels IV and V as proposed by Andersen et al. [17] and used in a previous study by Gincota et al. [18].

Contractures in the joints of the lower (hip, knee, ankle) and upper limbs (wrist) were described as present or absent by the neurologists or physical therapists, and represent a unique muscle adaptation where increased passive muscle stiffness causes reduced range of motion without active force production of the muscle [20]. Common criteria for categorising contractures and reduced range of motion are described by the use of “traffic light” categories in the Cerebral Palsy Follow-up Program in the Scandinavian countries (www.cpup.se/CPUPCriticalvalues_Children).

The only available socio-economic data were level of income and place of residence.

### Associated impairments

Associated impairments include seizures, cognition, communication, feeding, hearing, and visual disorders. Speech therapists described speech and feeding abilities in free text in the medical records. Speech was classified on a five-level scale from zero to four, where zero indicated normal speech, level I indicated indistinct speech, level II obviously indistinct speech, level III severely indistinct speech (difficult to understand) and level IV indicated children with no speech. Cognitive function was assessed using the Development Assessment of Young Children Evaluation tool (DAYC) and classified into intellectual disability (IQ score < 70) and normal intellectual ability (IQ score ≥ 70) [21].

### Statistical analyses

Descriptive statistics were used to generate frequencies and central tendencies. A flow chart illustrates the inclusion of the study sample. Comparisons between children with CP who were and were not enrolled in EI and/or FU services regarding subtype of CP, GMFCS and BFMF level, GA, BW, and level of income were performed using chi square tests.

Odds ratios (OR) and their associated 95% confidence intervals (CI) were calculated by means of two logistic regression models. In the first model receiving EI and/or FU served as dependent variable, while GMFCS level, BFMF level, GA and level of income served as independent variables (Table 4) to investigate which subgroups of children that were more likely to be enrolled into the early intervention and/or the follow-up program .. In the second model, having muscle contractures or not is the dependent variable, and being enrolled into the EI and/or FU-programs, GMFCS and BFMF levels, GA, and level of income were the independent variables. This model investigated potential protective impact of early intervention on development of muscle contractures in the children with CP.

Data analyses were conducted using the Statistical Package for Social Sciences for Windows version 22.0 (SPSS Inc., Chicago, IL, USA). A significance level of 0.05 was chosen.

### Ethics

Ethical approvals and collaboration agreements were obtained and formalised by all involved institutions.

Parents or primary caregivers of patients treated at this centre signed an informed consent form stating that medical data collected as part of the admission may be used for research purposes. The use of this regularly collected medical information in the present study was approved as described above and did not require a new individual consent form to be completed.

## Results

Among all 351 children born in 2009–2010 diagnosed with CP, mean age at diagnosis was 4.7 years and 166 (47%) had been enrolled in EI and/or FU programs. Of those 166 children, 112 (67%) were included in a FU program at the Institute of Mother and Child, 95 (85%) from rural areas. The remaining 54 children (33%) were included in EI at CEI Voinicel (Table 1).

**Table 1:** Enrollment into early intervention (EI) or follow-up program (FU) related to subtype of cerebral palsy (CP) according to surveillance of cerebral palsy in Europe (SCPE)

Subtype of CP according to SCPE	No Intervention N (%)	EI and/or FU program N (%)	Total N (%)	*P* value
Spastic Unilateral	70 (77)	21 (23)	91 (100)	.023
Spastic Bilateral	79 (40)	117 (60)	196 (100)	
Dyskinetic	18 (47)	20 (53)	38 (100)	.263
Ataxic	15 (71)	6 (29)	21 (100)	.002
Not Classified	3 (60)	2 (40)	5 (100)	.071
Total	185 (53)	166 (47)	351 (100)	

Among the 166 children enrolled in EI and/or FU programs, 84 (35%) children were classified at GMFCS levels I–III, compared to 157 (65%) of the children who had no intervention. However, among those enrolled in EI and/or FU programs, 82 children (74%) were classified at GMFCS level IV-V, compared to 28 children (26%) who were not enrolled (Table 2).

**Table 2:** Enrollment into early intervention (EI) or follow-up program (FU) related to gross- and fine motor function, gestational age, birth year and level of income

Gross- and fin motor function and perinatal data categories	No Intervention N (%)	EI and/or FU program N (%)	Total N (%)	*P* value
GMFCS^a^ categories I-III, IV-V				
Level I, II, III	157 (65)	84 (35)	241 (100)	.001
Level IV, V	28 (26)	82 (74)	110 (100)	
Total	185 (53)	166 (47)	351 (100)	
BFMF^b^ categories I-II, III, IV-V				
I-II	122 (68)	56 (32)	178 (100)	.001
III	37 (47)	42 (53)	79 (100)	
IV-V	26 (28)	68 (72)	94 (100)	
Total	185 (53)	166 (47)	351 (100)	
GA^c^ in categories				
< 28 GW	1 (6)	16 (94)	17 (100)	.042
28–31 weeks of gestation	4 (12)	30 (88)	34 (100)	
32–36 weeks of gestation	27 (54)	23 (46)	50 (100)	
> 37 weeks of gestation	153 (61)	97 (39)	250 (100)	
Total	185 (53)	166 (47)	351 (100)	
Birth weight				
< 1000 g	1 (8)	11 (92)	12 (100)	.038
1000-1499 g	4 (15)	23 (85)	27 (100)	
1500-2499 g	32 (45)	39 (55)	71 (100)	
> 2500 g	148 (61)	93 (39)	241 (100)	
Total	185 (53)	166 (47)	351 (100)	
Year of birth				
2009	77 (50)	78 (50)	155 (100)	.053
2010	108 (55)	88 (45)	196 (100)	
Level of income				
Low	62 (52)	58 (48)	120 (100)	.433
Medium and high	123 (53)	108 (47)	231 (100)	
Total	185 (53)	166 (47)	351 (100)	

^a^*GMFCS* Gross motor function classification system

^b^*BFMF* Bimanual fine motor function

^c^*GA* Gestational age

A significantly larger proportion of children with severely impaired hand function (BFMF IV and V) were enrolled in EI and/or FU programs: 68 (72%) of 94 children, compared to 98 children (38%) of 257 with BFMF level I–III (*p* = 0.001) (Table 2).

Nearly all the extremely preterm born children, 46 (90%) of 51 with GA ≤31 weeks, were enrolled in EI and/or FU programs, whereas just 97 (39%) of 250 children with GA ≥37 weeks were enrolled (*p* = 0.042) (Table 2).

No difference was found between 78 (50%) of 155 children born in 2009 and 88 (45%) of 196 children born in 2010 (*p* = 0.053) regarding enrolment in EI and/or FU programs (Table 2). Both socially disadvantaged families and those who belonged to middle- and high-income families were enrolled into the EI and/or FU programs, 52% versus 48%, respectively (Table 2).

The results of the second step of the study did not show statistically significant differences between outcomes in any of the developmental areas (intellectual development, speech, feeding abilities, and gross or fine motor function) between the groups enrolled and not enrolled in the EI and/or FU programs (Tables 2 and 3).

**Table 3:** Comparison of associated and secondary impairments outcomes between children enrolled or not enrolled in early intervention (EI) and/or follow-up (FU) programs

Associated impairments	No Intervention N (%)	EI and/or FU program N (%)	Total N (%)	*P* value
Intellectual impairments				
No	108 (63)	64 (37)	172 (100)	.54
Yes	77 (43)	102 (57)	179 (100)	
Total	185 (53)	166 (47)	351 (100)	
Speech impairments				
No	54 (67)	27 (33)	81 (100)	.059
Mild impaired	80 (61)	52 (39)	132 (100)	
Severe impaired	51 (37)	87 (63)	138 (100)	
Total	185 (53)	166 (47)	351 (100)	
Visual impairments				
No	156 (58)	113 (42)	269 (100)	.078
Yes	29 (35)	53 (65)	82 (100)	
Total	185 (53)	166 (47)	351 (100)	
Contractures				
No	121 (46)	144 (54)	265 (100)	.001
Yes	64 (74)	22 (26)	86 (100)	
Total	185 (53)	166 (47)	351 (100)	

Multivariable logistic regression analyses, where the enrollment into the EI and FU services is the dependent variable, showed that children at GMFCs levels IV-V had a greater chance to be enrolled into EI and FU services, almost 6 times more often compared to those at GMFCS levels I-III (OR: 5.566 95% CI 2.109–14.689), *p* = 0.000 (Table 4).

**Table 4:** Odds ratio (OR) and confidence intervals (CI) for enrolment into EI and/or FU programs for children with CP adjusted for gross motor function (GMFCS), hand function (BFMF), gestational age (GA), and level of income

EI and FU services					
Enrolled	166				
Not enrolled	185				
Total	351				
OR adjusted separately for:	Nr.	*p*-value	OR	95% C.I. Lower	95% C.I. Upper
GMFCS:					
Level I, II, III	241		Reference		
Level IV, V	110	.002	5.566	2.109	14.689
BFMF:					
Level I, II, III	254		Reference		
Level IV,V(1)	97	.719	1.202	.442	3.272
Income					
Medium and High income	231		Reference		
Low Income	120	.901	.969	.586	1.601
GA					
≥ 37GW	250		Reference		
< 37 GW	101	.000	4.227	2.479	7.208
Nagelkerke R Square	0.271				

In regards of gestational age, premature born children (< 37 GW) had four times increased chance of being enrolled into the EI and FU services compared to children born at term (≥37GW) (OR = 4.227, 95% CI 2.479–7.208), *p* = 0.000) (Table 4).

No statistical significant difference was found among the two groups of children enrolled and not enrolled into the EI and FU services according to BFMF levels (OR = 1.202, 95% CI 442–3.272) *p* = 0.719 and income levels of the families (OR = 0.969, 95% CI 0.586–1.601), *p* = 0.901 (Table 4).

Multivariable logistic regression analyses of risk of developing contractures in upper extremities (wrists) and lower extremities (hips, knees, ankles) were performed among children who were enrolled in EI and/or FU programs. We found that there was an increased risk (OR = 10.931, 95% CI 2.328–51.328, *p* = 0.002) for contractures among those non-walking children (GMFCS IV–V) not enrolled in EI and/or FU programs compared to those enrolled (Table 5).

**Table 5:** Odds ratio (OR) and confidence intervals (CI) of risk of developing contractures in children with CP enrolled or not enrolled into EI and/or FU programs adjusted for gross motor function (GMFCS), hand function (BFMF), gestational age (GA), and level of income

Contractures					
Yes	140				
No	211				
Total	351				
OR adjusted separately for:	Nr.	*p*-value	OR	95% C.I. Lower	95% C.I. Upper
GMFCS:					
Level I, II, III	241		Reference		
Level IV, V	110	.002	10.931	2.328	51.328
BFMF:					
Level I, II, III	254		Reference		
Level IV, V	97	.359	1.772	.521	6.020
Income:					
Medium and High income	231		Reference		
Low Income	120	.594	1.157	.676	1.981
GA					
≥ 37GW	250		Reference		
< 37 GW	101	.035	.510	.272	.954
Early Intervention and Follow Up program					
Yes	166		Reference		
No	185	.000	.020	.006	.067
Nagelkerke R Square	0.433				

No statistical significant difference in development of contractures was found regardless of hand function subgroup (BFMF) and income level of the families (Table 5).

The risk of developing contractures were decreased among children born preterm (< 37 GW) and enrolled into the EI and Fu programs, compared to those not enrolled (OR = 0.510, 95% CI 0.272–0.954), *p* = 0.035 (Table 5).

EI and FU was found to be a significant protective factor against developing contractures (OR = 0.020, 95%CI 0.006–0.067), *p* = .000 (Table 5).

## Discussion

In this case control study of EI and FU programs for children with CP in Moldova, we found that less than half of eligible children were enrolled in such programs. These results are comparable with the study by Scherzer et al. from 2012 and the WHO report (2012) in regards of global perspectives on early diagnosis and intervention for children with disabilities in Low and Middle Income Countries (LMIC) [22, 23].

The FU program at the Institute of Mother and Child was established in 2008, and the reference system was not established yet. This may contribute to explain the results of the first step of this study, showing low enrollment in the programs. Moreover, the specialists (physical therapists and paediatric neurologists) employed at the hospital department did not have the necessary evaluation tools and updated information.

Given that Institute of Mother and Child is a Republican hospital, most of the children across the country with severe disabilities are referred to it, and we assume that the biggest group of children with CP will receive services at this hospital, while the CEI Voinicel and two other centres (Tony Hawks Rehabilitation Centre and Republican Centre for Child Rehabilitation) cover Chisinau municipality.

The largest proportion of enrolled children was in those born extremely preterm (with GA ≤31). This significant difference between the GA groups referred and not referred to EI and/or FU programs (preterm and term birth) could be explained by the fact that the FU program is based in the main hospital for children. Almost 90% of all children born preterm were referred directly from the maternity ward to the FU program, because it was a new entity at the hospital, established in 2008.

This may also be explained by the fact that children born after complicated deliveries (including severe prematurity) are referred from all over the country to the Institute of Mother and Child for diagnosis and treatment, so they were included in the FU program to prevent possible complications. However, only a very small proportion of children born at term, with neurological symptoms, were referred to the FU program. This is to our knowledge the first study showing the inclusion of children born at term at risk for neurological adverse outcomes to FU programs and its outcomes in time.

The proportion of children with unilateral, ataxic and non-classified CP referred to EI and FU programs was significantly lower than those with more severe impairments. This might be explained by the mild clinical symptoms in the early life stages, as well as an insufficient level of competence in diagnosing unilateral CP among health care professionals, as it is stated in several publications (4,7,10). The use of the SCPE-recommended classification tree and inclusion and exclusion criteria could improve the number of children who would benefit from the EI or FU programs.

One positive finding of the present study is that accessibility of both the EI and FU services was equal to families regardless of social status (level of income), as well as place of residence (rural or urban) (Table 2). This might be explained by the fact that the Institute of Mother and Child is a Republican hospital, but EI was only offered to families from Chisinau municipality, because CEI Voinicel was the only EI centre in Moldova in 2009–2012.

The results of the second step of the present study are in line with the results of international research, showing no statistically significant positive impact of EI services on the associated impairments of CP [9–11]. We found no difference in motor or associated impairment outcomes between the groups enrolled or not enrolled in EI and/or FU programs. These results are similar to the studies in the systematic review by Morgan et al. [7] and the Cochrane review by Spittle et al. [10], stating that little evidence on enhancing the motor development was found. According to Mahoney et al., accelerated motor development or improved quality of movement beyond what could be expected from maturation could not be proved [24]. One important limitation of this study is that we did not include outcome measures such as Gross Motor Function Measure (GMFM), and therefore it was difficult to judge if there was an improved motor function in those enrolled in EI and/or FU programmes. However, we found that the risk of having contractures was increased more than 11 times among non-walking children with CP (GMFCS levels IV-V), when not being enrolled in EI and/or FU programmes, which confirms the results of the study by Skalsky & McDonald [25]. When we stratified by GA categories, the results showed that among preterm babies, early rehabilitation was a significant protective factor regarding development of contractures. This may contribute to enhance development of gross and fine motor function as documented in two new longitudinal studies from Norway based on the national CP-follow up program, showing that contractures have negative impact on gross motor development, and that intensive physiotherapy enhances gross motor development beyond what can be expected from maturation [26, 27].

These results may suggest that the interventions both in FU and in EI programs contribute to preventing development of contractures, comparable to the results obtained by Skalsky & McDonald [25]. Prevention of contractures is important to children’s possibilities for participation in everyday activities and play, as well as exploration and interaction with the physical and social environment [27].

No statistically significant difference was found in cognitive functioning, gross and fine motor function or speech between the children enrolled or not enrolled in EI and/or FU programs, which are in line with the results of the study by Mahoney et al. [24]. The participants in the present study were very young and cognitive challenges become more evident with age, particularly in school age, because even if the children with CP improve, the non-disabled peers improve more.

To be able to provide the right child the right intervention at the right time, correct sub-diagnosis and classification of functional level is crucial. It is therefore important for the authorities to revise the National Clinical Protocol of CP. The process of identification, diagnosis and evaluation of children with CP is likely to improve referral to EI or FU programs during the first 3 years of their lives, which is crucial for the whole family of children with special needs [28].

This also involves the best evidence-based knowledge of CP for the health care professionals involved in diagnosis and treatment of children with CP in Moldova. Revising the curricula of continuous medical education provided by the State Medical and Pharmacy University, ‘N, Testimitanu’, would have a high impact.

The results of the present study should be treated with caution because of the inclusion bias, where only the most severe cases were referred to the EI and FU programs.

## Conclusion

In Moldova, EI and FU programs seem to be offered mostly to extremely preterm children with CP, as well as to more severely impaired children, likely due to competence and capacity issues. Six centres are now providing early rehabilitation services in the central and northern parts of the country regions, compared to two at the time of the study. However, since only half of eligible children were enrolled in EI or FU programs, there is still a need for more centres in all 36 counties of Moldova, in order to improve geographical access to these services.

Based on the results of the present study, we would also recommend paying more attention to enrolling all preterm and term-born children with neurological signs in EI programs, as those enrolled showed a significantly lower prevalence of contractures, which may enhance functional development.

Revision of the National Clinical Protocol of CP with standardised and evidence-based instruments is necessary to secure early and accurate identification, diagnosis and evaluation of children with CP. To attain this, revision of the curriculum of continuous medical education provided by the State Medical and Pharmacy University, ‘N, Testimitanu’, would also be important.

## Data Availability

The datasets generated and analysed during the current study are not publicly available due to the restrictions to share the data to the third parties, based on the Management Committee of the Institute of Mother and Child decision -the hospital which provided the data (written permit from the Director of the hospital, where its specified that its forbidden to share the data to the third parties), but are available from the corresponding author on reasonable request.

## Abbreviations

BFMF: Bimanual fine motor function
BW: Birth weight
CP: Cerebral palsy
EI: Early intervention
FU: Follow-up program
GA: Gestational age
GMFCS: Gross motor function classification system
IQ: Intellectual quotient
SCPE: Surveillance of Cerebral Palsy in Europe

## References

[1] Rosenbaum P, Paneth N, Leviton A (2007). A report: the definition and classification of cerebral palsy April 2006. Dev Med Child Neurol.

[2] Surveillance of Cerebral Palsy in Europe (2000). Surveillance of cerebral palsy in Europe: a collaboration of cerebral palsy surveys and registers. Dev Med Child Neurol.

[3] Shepherd R (2014). Cerebral palsy in infancy.

[4] Novak I (2014). Evidence-based diagnosis, health care, and rehabilitation for children with cerebral palsy. J Child Neurol.

[5] Shonkoff JP, Meidels SJ (2000). Handbook of early childhood intervention.

[6] De Graaf-Peters VB, Hadders-Algra M (2006). Ontogeny of the human central nervous system: what is happening when?. Early Hum Dev.

[7] Morgan C, Darrah J, Gordon AM (2016). Effectiveness of motor interventions in infants with cerebral palsy: a systematic review. Dev Med Child Neurol.

[8] Guralnick MJ (2012). Preventive interventions for preterm children: effectiveness and developmental mechanisms. J Dev Behav Pediatr.

[9] Ravn IH, Smith L, Lindemann R (2011). Effect of early intervention on social interaction between mothers and preterm infants at 12 months of age: a randomized controlled trial. Infant Behav Dev.

[10] Spittle A, Orton J, Anderson PJ, Boyd R, Doyle LW (2015). Early developmental intervention programmes provided post hospital discharge to prevent motor and cognitive impairment in preterm infants. Cochrane Database Syst Rev.

[11] Blauw-Hospers CH, Hadders-Algra M (2005). A systematic review on the effects of early intervention on motor development. Dev Med Child Neurol.

[12] http://statbank.statistica.md/pxweb/pxweb/en/30%20Statistica%20sociala/?rxid=b2ff27d7-0b96-43c9-934b-42e1a2a9a774. Accessed 14 Apr 2019.

[13] Puiu I, Iatco C. Elaboration of the model of early intervention services for children with disabilities in Republic of Moldova. http://www.cnaa.md/en/thesis/8655/

[14] National Program “Strengthening perinatal healthcare in the Republic of Moldova” consolidated by Government Decision no. 1171 of October 18, 1997 and Ministry of Health no. 58 of 25.02.1998 (1998-2002). http://www.euro.who.int/__data/assets/pdf_file/0006/178053/HiT-Moldova.pdf. Accessed 14 Apr 2019.

[15] World Health Organisation (1992). International statistical classification of diseases and related health problems, 10th revision (ICD-10).

[16] Palisano R, Rosenbaum P, Walter S, Russell D, Wood E, Galuppi B (1997). Development and reliability of a system to classify gross motor function in children with cerebral palsy. Dev Med Child Neurol.

[17] Andersen GL, Irgens LM, Haagaas I, Skranes JS, Meberg AE, Vik T (2008). Cerebral palsy in Norway: prevalence, subtypes and severity. Eur J Paediatr Neurol.

[18] Gincota EB, Andersen GL, Vik T, Jahnsen R (2018). Cerebral palsy in Moldova: subtypes, severity and associated impairments. BMC Pediatr.

[19] Beckung E, Hagberg G (2002). Neuroimpairments, activity limitations, and participation restrictions in children with cerebral palsy. Dev Med Child Neurol.

[20] Smith LR, Lee KS, Ward SR, Chambers HG, Lieber RL (2011). Hamstring contractures in children with spastic cerebral palsy result from a stiffer extracellular matrix and increased in vivo sarcomere length. J Physiol.

[21] Simeonsson RJ, Rosenthal SL, Psychological & Developmental Assessment (2001). Development Assesment of Young Children (DAYC evaluation).

[22] Scherzer AL, Chhagan M, Kauchali S, Susser E (2012). Global perspective on early diagnosis and intervention for children with developmental delays and disabilities. Dev Med Child Neurol.

[23] Ertem IO, World Health Organization. Developmental difficulties in early childhood: prevention, early identification, assessment and intervention in low- and middle-income countries: a review: World Health Organization; 2012. https://apps.who.int/iris/handle/10665/97942

[24] Mahoney G, Rosenberg C, Fewell RR (2001). The effects of early motor intervention on children with down syndrome or cerebral palsy: a field-based study. J Dev Behav Pediatr.

[25] Skalsky AJ, McDonald CM (2012). Prevention and management of limb contractures in neuromuscular diseases. Phys Med Rehabil Clin N Am.

[26] Størvold Gunfrid V., Jahnsen Reidun B., Evensen Kari Anne I., Romild Ulla K., Bratberg Grete H. (2018). Factors Associated with Enhanced Gross Motor Progress in Children with Cerebral Palsy: A Register-Based Study. Physical & Occupational Therapy In Pediatrics.

[27] Storvold GV, Jahnsen RB, Evensen KAI, Bratberg GH. Is increased physical therapy frequency associated with increased gross motor improvement in children with cerebral palsy? A national prospective cohort study. Disabil Rehabil. 2018. 10.1080/09638288.2018.1528635. 10.1080/09638288.2018.1528635 30444146

[28] Novak Iona, Morgan Catherine (2019). High-risk follow-up: Early intervention and rehabilitation. Handbook of Clinical Neurology.

